# The Thymus Question

**Published:** 1915-06

**Authors:** Charles W. Hennington

**Affiliations:** Rochester, N. Y.


### BUFFALO MEDICAL JOURNAL

Yearly Volume 70. JUNE, 1915 No. 11

### ORIGINAL ARTICLES

The right is reserved to decline papers not dealing with practical medical and surgical subjects, and such as might offend or fail to interest readers. Contributors are solely responsible for opinions, methods of expression and revision of proof.

### The Thymus Question

By CHARLES W. HENNINGTON, M. D., Rochester, N. Y.

Read before the Rochester Academy of Medicine, April 14, 1915

Although I may touch upon other points, the essential topic under discussion is to be the significance of the Thymus gland in Graves disease. My paper is based largely upon a study of the recent literature, particularly that of the last year. Of the various articles, I have been most influenced by those of Dr. W. S. Halsted which appeared in the Johns Hopkins Hospital Bulletin, August, 1914, and February, 1915. This Thymus problem is still a very new subject. For this reason, as well as because of its inherent interest, it forms a most fascinating theme.

Halsted quotes Meltzer's statement concerning the Thymus gland made in 1910 as follows: "With our present knowledge of the importance of the other ductless glands we are hardly justified in assuming that the Thymus is a worthless fetal remnant. But we have to acknowledge that as yet there are no reliable observations or experiments which indicate clearly that the Thymus has a function in post-uterine life." This was in 1910 and that year practically marks the beginning of our active study of the relation of the Thymus gland to the body economy and to the other ductless glands particularly. The material of this paper is all much more recent however than the date just cited. Only for completeness of introduction, however, does it seem desirable to recall a few facts from the early history of the subject as follows: v. Mikulicz (1895) called attention to the occurrence of enlarged Thymus in severe cases of exophthalmic goitre; and Rehn (1899) suggested that it might be well to attack the Thymus surgically in that disease; and Hart (1908) expressed the opinion that abnormal activity of the Thymus might of itself produce the clinical picture of Graves disease.

The real beginning of this subject of the relationship of the thymus to disturbed function of the thyroid is with Garré of Bonn in 1911 when he reported the first instance in which the thymus had been primarily removed for the cure of exophthalmic goitre. Garré was well aware of course of the fact that a persistent or revivified thymus had repeatedly been observed in Basedow's disease, yet he was astonished to learn from the statistics obtained by his assistant, Dr. Capelle, that a "thymus persistens hyperplastica" had been found in 95 per cent of the fatal cases, whether death was due simply to the severity of the disease, or occurred during the operation, or early after the operation. So impressed was Garré with these findings that he determined to test the effect of thymectomy in those cases in which there was good reason to believe that the thymus was enlarged. He soon had a series of three cases in which only a thymectomy was done and a number in which a thymectomy was combined with the operation upon the thyroid. It seems without doubt that a persistent thymus aggravates the symptoms of exophthalmic goitre and that the removal of the thymus will decrease the heart rate remarkably besides favorably affecting various general symptoms. This is quite in agreement with the proof of Klose that in the thymus substance there is a heart poison. Other surgical work along similar lines has been done by Klose, by Sauerbruch, by v. Haberer of Innsbruck, who now has a remarkable series of 21 cases, and in this country by Halsted of Baltimore.

Before getting too far into a consideration of the various clinical and surgical points involved, it seems wise briefly to refer to other research work on the thymus which has been done during the last three years. Perhaps even before entering upon this we might try to recall those anatomical facts regarding the thymus which should be well understood.

The thymus has attained its full size at the end of the second year when it ceases to grow and gradually dwindles. At puberty it has become very small and after that it continues to become still smaller but it is not correct to say that it disappears. If examined when its growth is most active it consists of two or three irregular lobes placed in close contact along the middle line in the mediastinum and in the neck, and extending at that time from the fourth costal cartilage as high as the thyroid gland. The thymus is irregular in shape, rather flat, and oblong. It is of a pinkish gray color, soft and lobulated on its surfaces. Structurally it is composed of numerous lobules held together by delicate areolar tissue. The whole gland is invested by a similar but denser capsule. Microscopically there are a great number of follicles consisting each of a medullary and a cortical portion which differ markedly. The cortex consists largely of lymphoid cells while the medulla has but few lymphoid cells and instead many granular cells and the concentric corpuscles of Hassall which form the most easily recognizable elements on looking over the slide. Its dual structure from different embryological tissues suggests diverse functions as well.

The size and weight of a normal thymus in adults is a matter with which we ordinarily are unfamiliar. Hammer gives the following figures: At 15 years: 37 grams, about 1 1-6 oz.; 20 years: 25 grams, about 5-6 oz.; 25 years: 24 grams, about 5-6 oz.; 30 years: 20 grams, about 2-3 oz. It should always be recalled that in any wasting disease whatever, the thymus reacts very quickly and intensely and shrinks nearly to the point of disappearance. This explains why we usually, in our ordinary autopsies, find a very small thymus or fail to find it at all, while in autopsies in those instances of sudden perhaps accidental deaths the thymus is not so small and is therefore found more frequently. Thus our decision as to what to call an enlarged thymus must be based on a knowledge of its normal weight for that age.

Our designation of a persistent or hyperplastic thymus however is not based on size or weight merely. Histologically a persistent thymus or more correctly a sub-involuted thymus is one having a histological structure corresponding to an age younger than the actual age of the patient. A hyperplastic thymus has these same characteristics with marked increase in the medullary portion and with degeneration and atrophy of the Hassall's corpuscles as well.

Simmonds gives the results of thorough pathological study of the thymus in operative and in fatal cases of exophthalmic goitre. The material consisted of 12 from operation and 22 from autopsy. His tabulations are very convincing indeed, giving the histological picture as well as age of the patient and weight of the gland. It can be summarized as follows: In one-half of the cases of hyperthyroidism there is a pathological thymus; and in three-quarters of the cases of Basedow's disease there is a pathological thymus.

Kocher in one publication states "In nearly 50 per cent. of all cases of Basedow's disease a tendency to hyperplasia or tardy involution of the thymus is evident." In a later publication he says 60 per cent. His opinion is based upon pathological studies and upon clinical study. He finds this hyperplasia more frequently a complication in his younger patients as follows: In the 2nd decade 80 per cent.; in the 3d decade

40 per cent.; in the 4th and 5th decade 30 per cent.; after the 5th decade 0 per cent.

Kocher has also made the interesting observation that in cured cases of exophthalmic goitre the thymic enlargement is rarely demonstrable. Certainly not over 7 per cent.

Klose, Kuttner and others incline to an extreme view that every case of Basedow's disease has thymus as well as thyroid involvement.

Hart and others hold the opinion that Basedow's disease at times may be produced solely by the thymus.

The physiology of the thymus as revealed by experimental work forms a most marvelous chapter. I refer to the feeding of tadpoles with thymus, thyroid, adrenal, liver, spleen, hypophysis, brain, pancreas, testis, ovary, etc., by Gudernatsch of Cornell, published in the American Journal of Anatomy, January, 1914. Of all these substances the results clearly prove that thymus greatly stimulates growth and thyroid stimulates differentiation. The tadpoles fed upon thymus get very large indeed in a short time but fail to metamorphose. Whereas those fed on thyroid remain quite small but quickly differentiate, that is develop their legs and lose the tail.

Other physiological experimentation has been done as follows: Fiore and Franchette have apparently demonstrated that it is possible to accelerate the involution of the thymus by injecting the serum of adults and to postpone involution with serum of young of the same species.

Matti, Klose and Busch thought they found certain changes produced by the experimental removal of the thymus especially as to the osseous system but their work is not confirmed.

Nordman who removed the thymus in eight sets of pups concludes that the thymus is not necessary to life even in growing individuals.

Howland, Park and McClure in Baltimore also take the view that the thymus is not a vitally essential organ though some of their animals at the end of six months were smaller.

Svehle, Bircher, Bayer, Basch and Gebele in their work have demonstrated that the thymus and thyroid possess in common certain fundamental physiological properties.

Bircher for instance has twice produced typical Basedow's pictures by intra-peritoneal implantation of fresh pathologically hyperplastic thymus. Gebele similarly has implanted normal thymus into thyroidectomized dogs and thereby prevented cachexia strumipriva. Gudernatsch with his tadpoles cited above and other experimenters have proven that there are antagonistic factors at work between the thymus and thyroid and with other glands of internal secretion as well especially adrenal, ovary and possibly others.

It does not seem likely however that the physiology of the thymus will be worked out completely in the laboratory. It is largely a clinical problem which finally will be solved by a study of patients. Thus far considerable progress has been made as indicated by the studies cited earlier in this paper.

Looking at the question from a practical medical standpoint the crux of the problem seems to be just this: In which cases of exophthalmic goitre shall we suspect thymic enlargement? and of what advantage is it to us to recognize this condition?

Perhaps the greatest advantage in differentiating these cases of exophthalmic goitre complicated by enlarged thymus is that we will recognize them as the more severe and dangerous cases, and therefore give them the more careful thought in their management. These are the cases that may require a several-stage operation even though they might not appear to be in such an extreme state on ordinary clinical observation. These are the cases that are more likely to be associated with a rapid onset or with periods of rapidly advancing recrudesences and with tremendous loss in weight, etc., and which it has been recognized for some time past are unfavorable operative risks in this early stage of the disease. These cases should always have a thymectomy in addition to their thyroid operation. The thymus operation will cause the greatest improvement in their heart symptoms both objective but especially subjective. Thymectomy is an operation not too difficult for those thoroughly trained as surgeons. But better still, these are the cases that if recognized should have preliminary treatment of the thymus with Roentgen rays. Such treatment consists of a series of six exposures on successive days followed after a month by a second and third series. In fact some will be cured by this alone. Most of them will experience the most favorable effect on the heart. Some of the sad cases which have had one or more operations upon their thyroids but without cure, may be helped greatly by X-Ray treatment of the thymus. This latter statement is based upon Halsted's series of cases of this type. This favorable experience with the X-Ray treatment of enlarged thymus would seem to condemn any effort at operative removal of the thymus. This is true to a degree in which the condition is diagnosed clinically. But if the thymus is not diagnosed until at the operation itself, then of course an immediate thymectomy is indicated. This search for thymus tissue in the mediastinum is done by opening the fascia at the root of the neck and drawing up the contents of the upper mediastinum just back of the manubrium. It should form a routine part of every thyroid operation for Graves disease. Finally an immense advantage lies in recognizing a thymus in the preliminary clinical study of those more obscure cases of thyroid derangement associated with probable polyglandular involvement. A case of this nature has had my attention for a year past. Such polyglandular cases may be largely of a thymus type with a preponderance of distress due to subjective heart symptoms and possibly respiratory distress. It is likely that complete relief may be afforded them by treating the thymus with X-Rays.

The next practical medical question to arise is this: When shall we suspect the presence of an enlarged thymus? And how shall we proceed to prove its presence? Although there is still much doubt as to what symptoms are suggestive of thymus involvement yet I think that it is safe to suspect a thymus when we have a preponderance of those symptoms referable to the vago-tonic (autonomic) in contra-distinction to the sympathetico-tonic nervous system. Our attention is therefore directed to the following: Pronounced heart distress with relatively moderate tachycardia, sweating, diarrhoea, lacrimation, only slight exophthalmos, small goitre, emaciation sometimes extreme, grayish bronzing of the skin, and above all dyspnoea or a sense of pressure in the chest. Our suspicion might be increased if the Kocher blood picture of a relative lymphocytosis was marked, or if the patient were to react definitely to the vago-tonic tests such as pilocarpin stimulation, atropin depression, and remain refractory to adrenalin which stimulates the sympathetic.

But without performing these delicate and possibly distressing tests we may proceed at once to prove the presence of an enlarged thymus by percussion and by X-Ray. Enlargement is looked for especially to the left border of the manubrium. The percussion note need not be dull but merely impaired. The note may change rhythmically with forced respiration or swallowing depending upon the change of position of the thymus, it being drawn up because of its fascial attachment to the thyroid and so to the trachea and larynx. These patients may have distress on over-extending the neck and on swallowing in this attitude. Sometimes they have distress when pressure is made at the root of the neck just back of the manubrium in a direction downward toward the mediastinum. Our main reliance is on the X-Ray examination however. The plate may show the thymus when the fluroscope does not. If the fluroscope shows it also then it is essential to note whether the position changes on swallowing as just explained, and with forced respiration but this later observation is rather unsatisfactory. The shadow on the plate usually has faint indistinct borders and is quite separate from the rest of the mediastinal shadow.

Thus we have considered the Thymus Question with special reference only to exophthalmic goitre. There are other phases which we cannot touch upon. These include such clinical problems as thymic asthma (which seems to be not so much mechanical as an instance of hyperthymisation) thymic death, status thymo-lymphaticus and other states probably associated with a hypoplasia of the thymus, and the general problem of the interrelation of ductless glands and the resultant complex of polyglandular disease.

292 Alexander Street, Rochester, N. Y.

---

**Typhoid.** Emile Weil, Presse Med., Jan. 14, 1915, advocates the drop method of rectal injection of Murphy, using 1 liter of water containing 50 grammes of glucose or cane sugar in solution, at 40 degrees, introduced in the course of three or four hours. The results are said to be equal to the cold bath treatment, in reduction of temperature, apparently from detoxication and increased nutrition.

E. Sergent, ibid., Dec. 31, 1914, considers the adynamia of typhoid as largely due to suppression of suprarenal secretion and, therefore, administers 1/4-1/2 m. g. twice daily either in ampoules of 1 C.C. or diluted with 250 C.C. of physiologic salt solution. (Note: In 1900, we used adrenalin quite extensively as a general cardiac and circulatory tonic, almost to the exclusion of digitalis, strophanthus, etc., with very good results but discontinued the treatment on account of the warnings—probably exaggerated—that adrenalin might produce pancreatic diabetes. Ed.)

Widal, Abrami and Etienne Brissaud, Bull. de l'Acad. de Med. Feb. 16, 1915, advocate the use of the patient's own serum, diluted and administered in increasing doses.

---

**Vital Statistics of New York State.** From the Health Bulletin, it appears that the death rate is decidedly in favor of places of large population, 14:1000 for Greater N. Y., 14.3 for Rochester, 15.5 for Buffalo, 16.2 for cities of 50,000 to 175,000, 15.3 for cities of 20,000-50,000, 16.1 for cities of 10,000-20,000, 16.3 for cities under 10,000, 14.8 for rural communities. Various high death rates are explained by the presence of large institutions, penal, for the insane, paupers, etc. Typhoid kills about 8 2/3 per 100,000 there being not much difference between rural and urban rates but marked individual fluctuations. Pulmonary tuberculosis still kills about 1 person in 10, as for many years, indicating, we think, in spite of some pessimistic opinions, that its prevention and cure have progressed almost exactly as much as for disease generally.

---

**300 Premature Births in One Day** is one of the most terrible results of the invasion of Belgium. Prof. Jacobs of the University of Brussels vouches for the number, Med. Rec. Dec. 19, 1914.

